# Supplementary material for: Antifibrotic TSG-6 Expression Is Synergistically Increased in Both Cells during Coculture of Mesenchymal Stem Cells and Macrophages via the JAK/STAT Signaling Pathway
Source: Int J Mol Sci. 2022 Oct 28;23(21):13122. doi: 10.3390/ijms232113122 (PMC9656625; doi:10.3390/ijms232113122)
Supplement: Supplementary file 1 [file ijms-23-13122-s001.zip › ijms-1954512-supplementary.pdf]

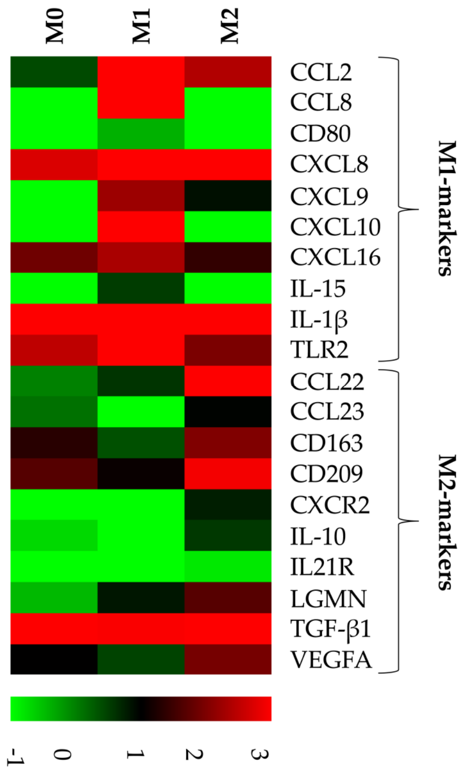

Supplementary Figure S1. Heat map analysis of next-generation sequencing (NGS) data obtained from M0, M1, and M2 macrophages. THP-1 monocytes were differentiated into macrophages (M0 phase) using TPA (100 nM) for two days. M0 macrophages were washed with PBS and then transdifferentiated into M1 or M2 phase by treatment with IFN-γ (20 ng/mL) and LPS (10 pg/mL) or 20 ng/mL each of IL-4 and IL-13 for two more days, respectively. NGS proceeded according to the method mentioned in Kim S. et al. [1]. The analysis was conducted by Theragen (Seongnam, Korea). Results show that M1 or M2 markers were significantly increased in M1 or M2 macrophages, respectively.

1. Kim, S.; Jung, P.Y.; Lee, J.S.; Hwang, S.; Sohn, J.H.; Yoon, Y.; Bae, K.S.; Eom, Y.W. Cultured human skeletal muscle satellite cells exhibit characteristics of mesenchymal stem cells and play anti-inflammatory roles through prostaglandin E2 and hepatocyte growth factors. *Cell Biol Int* **2021**, *45*, 2443-2451, doi:10.1002/cbin.11688.
